# Microfluidic-prepared DOTAP nanoparticles induce strong T-cell responses in mice

**DOI:** 10.1371/journal.pone.0227891

**Published:** 2020-01-24

**Authors:** Yasunari Haseda, Lisa Munakata, Jie Meng, Ryo Suzuki, Taiki Aoshi

**Affiliations:** 1 Vaccine Dynamics Project, BIKEN Innovative Vaccine Research Alliance Laboratories, Research Institute for Microbial Diseases, Osaka University, Suita, Osaka, Japan; 2 Laboratory of Drug and Gene Delivery Research, Faculty of Pharma-Science, Teikyo University, Itabashi-ku, Tokyo, Japan; 3 Vaccine Dynamics Project, BIKEN Center for Innovative Vaccine Research and Development, The Research Foundation for Microbial Diseases of Osaka University, Suita, Osaka, Japan; National Institute of Animal Biotechnology, INDIA

## Abstract

For the induction of antigen-specific T-cell responses by vaccination, an appropriate immune adjuvant is required. Vaccine adjuvants generally provide two functions, namely, immune potentiator and delivery, and many adjuvants that can efficiently induce T-cell responses are known to have the combination of these two functions. In this study, we explored a cationic lipid DOTAP-based adjuvant. We found that the microfluidic preparation of DOTAP nanoparticles induced stronger CD4^+^ and CD8^+^ T-cell responses than liposomal DOTAP. The further addition of Type-A CpG D35 in DOTAP nanoparticles increased the induction of T-cell responses, particularly in CD4^+^ T cells. Further investigations revealed that the size of DOTAP nanoparticles, prepared buffer conditions, and physicochemical interaction with vaccine antigen are important factors for the efficient induction of T-cell responses with a relatively small antigen dose. These results suggested that microfluidic-prepared DOTAP nanoparticles plus D35 are a promising adjuvant for a vaccine that induces therapeutic T-cell responses for treating cancer and infectious diseases.

## Introduction

The induction of antigen-specific T-cell responses by vaccination has been shown to be protective against infectious diseases [1, 2] and cancer [3–5]. To induce antigen-specific T-cell responses, particularly by using protein antigen, there is a need to include an appropriate immune adjuvant. Immune adjuvants can be divided into two functional categories: immune potentiator and delivery system [6, 7]. Immune potentiators generally stimulate various innate immune pattern recognition receptors. Typical immune potentiators are pathogen-associated molecular patterns, which are mainly various toll-like receptor (TLR) agonists including poly I:C (TLR3), MPL (TLR4), and CpG (TLR9) [8–10]. Delivery systems feature various particles consisting of synthetic polymers, liposomes, and oil emulsions [11, 12]. However, many adjuvants that can efficiently induce T-cell responses are adjuvant combinations that exhibit both immune potentiator and delivery system functions, such as GSK adjuvant systems, ISCOMATRIX, and CAF family adjuvants [13–17]. Interestingly, most of these are composed of lipid plus immune stimulator.

Cationic lipids (such as 1,2-dioleoyl-3-trimethylammonium-propane [DOTAP]) have been used as antigen carriers for cancer vaccines and showed effective adjuvant activity by themselves [18–21]. Furthermore, combinations of a cationic lipid (such as dimethyldioctadecylammonium [DDA]) and a synthetic immune potentiator have been shown to be promising adjuvant combinations to induce both CD4^+^ and CD8^+^ T-cell responses [22, 23]. The cationic lipid DOTAP has also been used as a carrier of immune potentiators, such as CpG immunostimulatory oligodeoxynucleotide (ODN), to the endosomal compartment where the CpG receptor TLR9 resides [24, 25]. It has also been shown that the combination of DOTAP and CpG ODN enhanced CpG-mediated biological activities in vivo [26, 27].

The recently developed microfluidic method enables smaller (under 100 nm) formulations of lipid nanoparticles to be created [28–32]; this was not achievable using the conventional lipid film hydration method [33]. This microfluidic technology has been commonly applied to form siRNA–lipid nanoparticles, but its application for producing lipid-based vaccine adjuvants has not been widely examined.

In this study, we examined microfluidic-prepared DOTAP for the induction of T-cell responses against model protein antigens and compared the adjuvanticity among different preparations of DOTAP in mice. We found that the preparation method, size, and interaction with antigen are important factors influencing the adjuvanticity of DOTAP-based lipid particle adjuvant. We also demonstrated that the combination of DOTAP and Type-A CpG ODN is a simple and promising adjuvant for efficiently inducing both CD4^+^ and CD8^+^ T-cell responses against protein antigen.

## Materials and methods

### Materials

Low-endotoxin ovalbumin (OVA) was purchased from WAKO. D35 (g^gtgcatcgatgcagggg^g^g) and K3 (a^t^c^g^a^c^t^c^t^c^g^a^g^c^g^t^t^c^t^c) were purchased from GeneDesign (Osaka, Japan). C2395 (t^c^g^t^c^g^t^t^t^t^c^g^g^c^g^c^g^c^g^c^c^g) and P21889 (t^c^g^t^c^g^a^c^g^a^t^c^g^g^c^g^c^g^c^g^c^c^g) were synthesized by GeneDesign (Osaka, Japan); ^ indicates phosphorotihoate bonds. DOTAP was purchased from Lipoid GmbH. DOTAP liposomal transfection reagent (1 mg/mL) (Roche; Cat. No. 11 202 375 001) was purchased from Sigma. Hen egg lysozyme (HEL) was purchased from Sigma.

### Preparation of DOTAP particles by NanoAssemblr (DOTAP-Nano)

DOTAP-Nano was prepared with the NanoAssemblr Benchtop (Precision NanoSystems Inc., BC, Canada), which can mediate bottom-up self-assembly for nanoparticle synthesis with microfluidic mixing technology. Briefly, DOTAP was dissolved in ethanol. The lipid solution (10 mg/mL) in ethanol and 25 mM acetate buffer (pH 4.0) were injected into the microfluidic mixer at a 1:3 volume and at a combined final flow rate of 15 mL/min (3.75 mL/min ethanol, 11.25 mL/min aqueous). The DOTAP-Nano mixtures were immediately dialyzed (50 kDa MWCO dialysis tubing; Repligen Corporation, MA) against 5% glucose solution to remove ethanol. DOTAP-Nano was then concentrated to approximately 1.5–2.0 mg/mL DOTAP by using Amicon Ultra Centrifugal filters (100 kDa MWCO; Merck KGaA, Darmstadt, Germany) and sterilized through a 0.22 μm PVDF filter (Merck KGaA). The preparation of all lipid nanoparticles was performed at room temperature.

### Preparation of DOTAP particles by lipid film hydration method (DOTAP-film)

DOTAP was dissolved in chloroform (5–10 mg/mL). After drying to form a thin lipid film on the bottom of a round-bottomed flask, the lipid film was hydrated in 5% glucose, freezed/thawed five times and then sonicated once. Thereafter, lipid was filtered through a 0.45 μm PVDF filter (Merck KGaA).

### Physical parameter analysis of DOTAP particle preparation and antigen/adjuvant interaction

The size distribution was measured by dynamic light scattering (DLS), and the zeta potential was measured by particle electrophoresis with Zetasizer Nano-ZS90 (Malvern Panalytical Ltd., UK). A representative size distribution and polydispersity index of DOTAP-Nano, DOTAP-Lipo, and DOTAP-film with or without antigen and D35 were shown in S1 Fig. DOTAP-nano with B, C, P type CpG interaction was also evaluated by DLS and ultrafiltration. Representative results are shown in S2 Fig.

### Mice and immunization

Mice were purchased from CLEA Japan. Female C57BL/6 or BALB/c mice aged 4–10 weeks old were used for all experiments. All animal experiments were approved by the Animal Care and Use Committee of Osaka University. Mice were housed in a room maintained at constant room temperature (22–24°C) with a 12-hour-light/12-hour-dark cycle (lights on at 8:00 am, lights off at 8:00 pm) and had free access to food and water. The experiment was performed in accordance with the Regulations on Animal Experiments at Osaka University (Permit No. Biken-AP-H26-12-2). In most of the experiments, three mice per group (total 129 mice for immunization experiments presented in this study) were immunized with antigen (OVA, HEL: 10 μg) only or with antigen plus adjuvants (CpG ODNs: 10 μg, DOTAP-Nano/-Lipo: 100 μg). A mixture of DOTAP-nano/antigen and DOTAP-nano/antigen/CpG were prepared by simply adding each components in 5% glucose solution and vortexed. A mixture with a total volume of 100 μL per mouse was injected once at the tail base (50 μL right side and 50 μL left side) on day 0, and the mice were sacrificed for T cell response assay on day 7. For three times immunization experiment, the mice were immunized at the tail base with two weeks’ intervals such as immunization at day 0, day14, and day 28, and then the mice were sacrificed 7 days later of the last immunization such as day 35 for T cell and antibody response assays.

### Splenocyte preparation and in vitro stimulation for ELISA

The mice were sacrificed by cervical dislocation, and their spleens were collected; single-cell suspensions were prepared. Red blood cells were lysed with ACK lysis buffer (150 mM NH_4_Cl, 10 mM KHCO_3_, 0.1 mM Na_2_EDTA), and cells were washed with RPMI 1640 and suspended in R-10 medium (RPMI 1640 medium supplemented with 10% fetal calf serum, 100 units/mL penicillin, and 100 μg/mL streptomycin). Splenocytes were plated on 96-well flat plates at 2 × 10^6^ cells/well/200 μL. OVA257-264 peptide (SIINFEKL), OVA full protein, HEL107-116 peptide (AWVAWRNRCK), and HEL full protein at 5 μg/mL (final concentration) were added to the cell cultures and incubated at 37°C in a CO_2_ incubator. The overnight cultured supernatant was collected and subjected to mouse IFN-gamma ELISA (Mouse IFN-γ DuoSet ELISA kits [R&D Systems]).

### Bone marrow (BM) preparation

The femurs and tibiae of mice (total three mice for this BM experiment) were removed, and the surrounding muscle tissues were cut using scissors. The marrow was flushed out with RPMI 1640 by using a syringe with a 25G needle. The cells were treated with ACK lysis buffer for 5 min. After washing the cells once in RPMI 1640 and counting them, BM cells were suspended in R-10 medium.

### Human peripheral blood mononuclear cells (PBMCs)

PBMCs were prepared from healthy Japanese adult volunteers who had provided written informed consent to participate in this study. All experiments using human PBMCs were approved by the Institutional Review Board of the Research Institute for Microbial Diseases, Osaka University (Permit No. 26–5). After preparing PBMCs using Ficoll-Paque PLUS (GE) and LeucoSep (Greiner), they were washed twice with RPMI 1640 medium and resuspended in R-10 medium (RPMI 1640 medium supplemented with 10% fetal calf serum, 100 units/mL penicillin, and 100μg/mL streptomycin).

### In vitro stimulation and cytokine ELISA

Human PBMCs or mouse BM cells were plated on 96-well plates at 1 × 10^6^ cells/well/200 μL. D35 (1 μM = 6.3 μg/mL) and DOTAP-Nano/-Lipo or D35+DOTAP-Nano/-Lipo were added to the cell cultures overnight at 37°C in a CO_2_ incubator. The centrifuged supernatant was collected and used for cytokine ELISA. Human IFN-α was measured with human IFN-α pan-ELISA development kit (Mabtech). Mouse IFN-α/β (type I IFN) production was measured using B16-Blue IFN-α/β reporter cells (InvivoGen).

### Serum and antibody titer

Mice (n = 3 per group) were anesthetized with 3.0% isoflurane. Blood was collected using a heparin-coated microcapillary and centrifuged (6500 g, >20 min). The levels of OVA-specific antibodies in plasma were determined by ELISA. Ninety-six-well plates were coated with 100 μg/ml OVA or standard antibodies (total IgG: MBL, IgG1, IgG2c; Southern Biotech) in a carbonate buffer (pH 9.6). The wells were blocked with Blockace (KAC Co., Ltd., Japan), and diluted plasma from the immunized mice was incubated on the antigen-coated plates. After washing, goat antimouse total IgG-, IgG1-, or IgG2c-conjugated HRP (Southern Biotech) was added and incubated for 2 h at room temperature. After additional washing, the plates were incubated with TMB (KPL) for 5–15 min, the reaction was stopped with 2N H_2_SO_4_, and the absorbance at OD_450_ was measured.

### Ultrafiltration (UF)

Protein only or protein plus DOTAP-Nano in glucose and PBS buffer was loaded into Amicon Ultra Centrifugal filter units (100k) (Merck Millipore) and centrifuged at 14,000 g for 1 min. Thereafter, protein concentration in flow through (FT) was calculated by Qubit Protein Assay Kit (Thermo Fisher Scientific).

### Statistical analysis

The statistical significance of the differences was calculated using GraphPad Prism 6 software. The data are presented as mean ± SD, and unpaired *t*-test or one-way analysis of variance was used for statistical analysis. Significant differences are indicated with asterisks (*P < 0.05 and **P < 0.01).

## Results

### NanoAssemblr-prepared DOTAP nanoparticles (DOTAP-Nano) induced stronger T-cell responses than commercially available liposomal DOTAP (DOTAP-Lipo) as vaccine adjuvant

**Fig 1:**
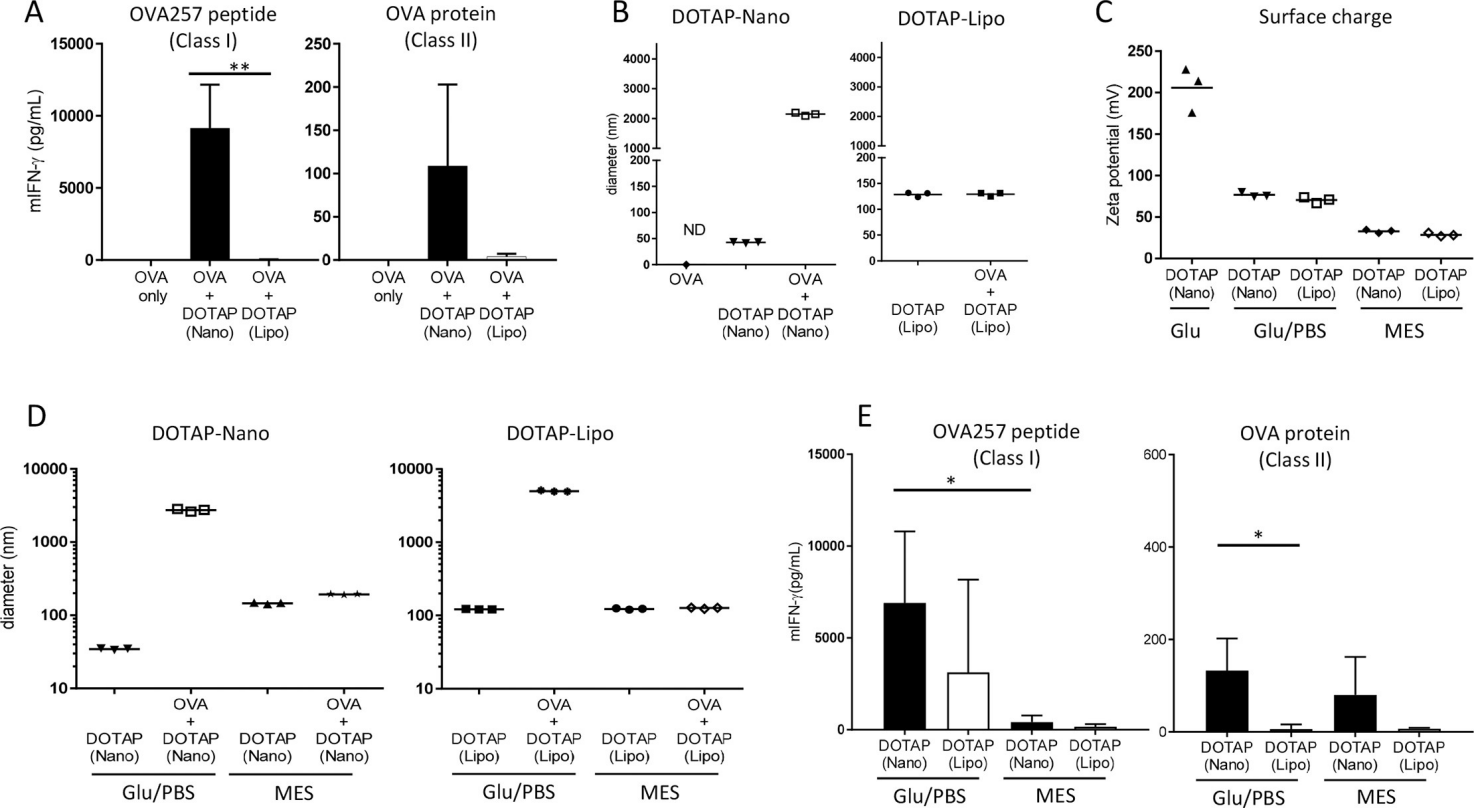
Particle preparations of DOTAP influenced the adjuvanticity. **(A)** C57BL/6J mice were immunized with OVA (10 μg) and DOTAP (100 μg) at the tail base. After seven days, splenocytes were collected and stimulated with OVA257-264 peptide, which induced an MHC class I–restricted CD8^+^ T-cell response, or with OVA protein, which induced an MHC class II–restricted CD4^+^ T-cell response. Twenty-four hours later, mouse IFN-gamma in the culture supernatant was determined by ELISA. The bar graph indicates the mean ± SD of three mice per group.(B) The sizes of OVA only, lipid particle only, and lipid mixed with OVA were determined by the dynamic light scattering (DLS) method. An increase in particle size indicates that the lipid particle interacts with OVA protein. Each dot indicates one measurement. (C) The zeta potential of lipid particles in the indicated buffer conditions was measured by a zetasizer. Each dot indicates one measurement. (D) The sizes of lipid particles and lipids mixed with OVA were measured by DLS under the indicated buffer conditions. An increase in particle size indicates that the lipid particle interacts with OVA protein. Each dot indicates one measurement. (E) C57BL/6J mice were immunized at the tail base with OVA mixed with either DOTAP-Nano or DOTAP-Lipo under the indicated buffer conditions. After seven days, splenocytes were stimulated with OVA257-264 peptide, which induced an MHC class I–restricted CD8^+^ T-cell response, and with OVA protein, which induced an MHC class II–restricted CD4^+^ T-cell response. Twenty-four hours later, mouse IFN-gamma in the culture supernatant was determined by ELISA. The bar graph indicates the mean ± SD of three mice per group.

First, we compared two formulations of cationic lipid DOTAP for the T-cell-inducing adjuvant activity against model protein antigen OVA in mice. One is referred to as “DOTAP-Nano,” which was prepared by microfluidic methods using the NanoAssemblr system in our laboratory, and the other is called “DOTAP-Lipo,” which is a commonly used reagent for transfecting nucleic acids and is commercially available from Sigma in a “ready-to-use” format. Mice were immunized at the tail base with OVA antigen (10 μg/mouse) mixed with either one of the two different preparations of DOTAP as adjuvant. Seven days later, the splenocytes were stimulated in vitro with OVA257-264 peptide to detect the OVA-specific MHC class I–restricted CD8^+^ T-cell responses and with whole OVA protein to detect the OVA-specific MHC class II–restricted CD4^+^ T-cell responses. Although these two DOTAP preparations were made from the same chemical molecule, the resultant inductions of T-cell response were surprisingly very different. DOTAP-Nano prepared with NanoAssemblr induced robust CD8^+^ T-cell responses (Fig 1A; left) and CD4^+^ T-cell responses (Fig 1A; right), whereas DOTAP-Lipo showed almost no adjuvant effect (Fig 1A).

We then investigated the mechanisms underlying this difference in adjuvant effect. In other cationic lipid DDA-based liposomal adjuvants, cationic charge-dependent antigen absorption has been reported to influence adjuvanticity [34]; thus, we examined the interaction between DOTAP particles and OVA antigens by DLS measurement. The size of DOTAP-Nano itself was approximately 50 nm, and the addition of OVA antigen into the DOTAP-Nano containing solution increased the size to approximately 2000 nm (Fig 1B; left), thus indicating that DOTAP-Nano interacted with OVA antigen and formed large lipid and protein complexes. On the contrary, DOTAP-Lipo itself showed a size of approximately 130 nm, and the addition of OVA did not cause any changes in size, thus indicating that DOTAP-Lipo did not interact with OVA antigen (Fig 1B; right).

These results suggested that the difference in adjuvanticity of the two DOTAP preparations arose from the difference in physical interaction between antigen and cationic lipid. Although many other factors can affect antigen–lipid physical interactions, the electrostatic interaction was expected to be the main factor because the isoelectric point of OVA is approximately 4.5 and because DOTAP is always cationic within a broad pH range. The electrostatic interaction is also known to be influenced by the buffer system. In the experiments mentioned above, DOTAP-Nano was prepared in 5% glucose solution, and DOTAP-Lipo was immersed in MES-buffered saline (pH6.2) by the manufacturer. Therefore, the experiments reported in Fig 1A and 1B were actually performed under different buffer conditions: DOTAP-Nano plus OVA immunization and DLS measurement were performed in the buffer conditions of a 1:1 mixture of 5% glucose and PBS (Glu/PBS), whereas DOTAP-Lipo plus OVA was performed in MES-buffered saline conditions.

To examine the effects of these differences in buffer conditions on the DOTAP–antigen interaction in detail, we prepared DOTAP-Nano in MES-buffered saline instead of 5% glucose solution and similarly exchanged the buffer of DOTAP-Lipo from MES-buffered saline to Glu/PBS by UF. The zeta potential of DOTAP-Nano in 5% glucose was approximately 200 mV (Fig 1C). For both DOTAP-Nano and DOTAP-Lipo prepared in Glu/PBS, the zeta potential was approximately 70 mV. In MES-buffered saline, both values were approximately 30 mV (Fig 1C). The zeta potentials changed in a manner dependent on the buffer conditions, but the two preparations of DOTAP showed very similar zeta potentials under the same buffer conditions (Fig 1C). We also examined the interaction between DOTAP and OVA antigen in Glu/PBS or MES-buffered saline. In Glu/PBS, both DOTAP-Nano and DOTAP-Lipo showed apparent interactions with OVA, but this was not the case in the MES-buffered saline (Fig 1D). Consistent with these interactions, both DOTAP preparations in Glu/PBS showed robust CD8^+^ T-cell responses (Fig 1E; left) but not in MES-buffered saline (Fig 1E; left). In the case of CD4^+^ T-cell responses, MES-buffer DOTAP-Nano showed slight reductions in CD4^+^ T-cell responses; however, a comparable amount of IFN-γ production was still detected as that in Glu/PBS (Fig 1E; right). Interestingly, even under the Glu/PBS buffer conditions, DOTAP-Lipo did not induce strong CD4^+^ T-cell responses (Fig 1E; right). These results suggested that the low adjuvanticity of DOTAP-Lipo for the induction of CD8^+^ T-cell responses was mainly derived from the lack of interaction with OVA antigen, which was likely caused by MES-buffer-dependent zeta potential reduction (Fig 1C and 1D). For the induction of CD4^+^ T-cell responses, NanoAssemblr-formulated DOTAP-Nano showed better adjuvanticity irrespective of the buffer conditions, thus suggesting that the adjuvanticity for the induction of CD4^+^ T-cell responses was not necessarily dependent on the interaction between adjuvant and antigen; instead, it may be dominantly dependent on the size of DOTAP particles. However, we could not rule out the possibility of the weak interaction between DOTAP-Nano and OVA in MES-buffered conditions (potentially reflected in the slight increase of size shown in Fig 1D) also influencing the adjuvanticity of DOTAP-Nano for the induction of CD4^+^ T-cell responses in MES buffer (Fig 1E).

### DOTAP-Nano induces stronger T-cell responses than the DOTAP prepared by the lipid film hydration method (DOTAP-film)

The abovementioned results showed that buffer conditions influenced the interaction between DOTAP and antigen and the resultant induction of T-cell responses. However, the effect of differences in lipid particle preparation on the adjuvanticity was still not clarified because DOTAP-Nano was prepared by ourselves and because DOTAP-Lipo had already been made in advance commercially. To directly compare the influence of the differences in preparation method on the adjuvanticity, we prepared “DOTAP-film” by using the same DOTAP powder as that in the DOTAP-Nano preparation in our laboratory. DOTAP-film was formed by the conventional lipid film hydration method, and it was directly compared with DOTAP-Nano prepared with NanoAssemblr.

**Fig 2:**
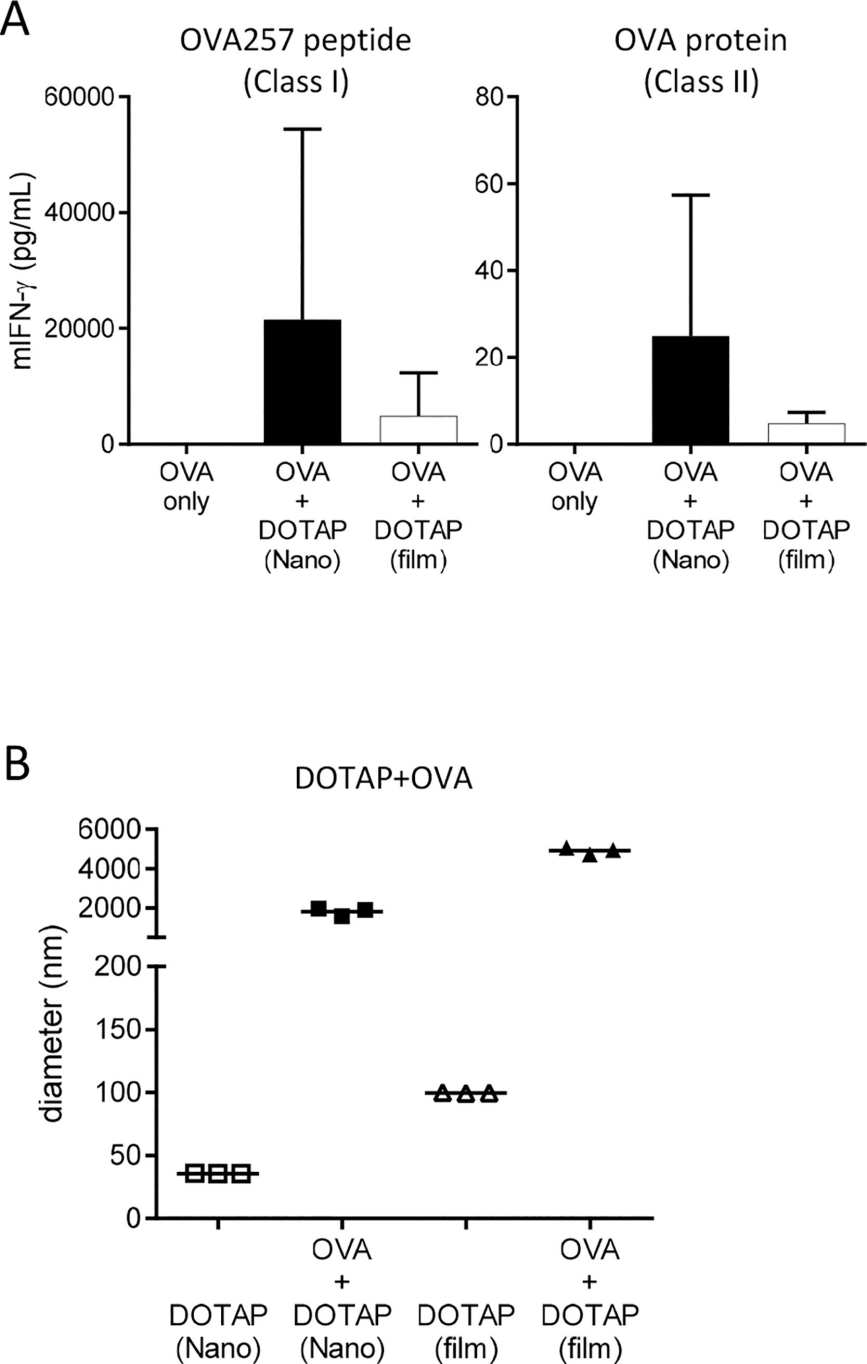
DOTAP-Nano showed better adjuvanticity than DOTAP-film preparation. **(A)** C57BL/6J mice were immunized with OVA (10 μg) and DOTAP (100 μg) at the tail base under the same buffer conditions of Glu/PBS. After seven days, splenocytes were collected and stimulated with OVA257-264 peptide and OVA protein. Twenty-four hours later, mouse IFN-gamma in the culture supernatant was measured by ELISA. The bar graph indicates the mean ± SD of three mice per group. (B) The sizes of lipid particles and lipids mixed with OVA were measured by DLS under Glu/PBS buffer conditions. An increase in particle size indicates that the lipid particle interacts with OVA protein. Each dot indicates one measurement.

Either DOTAP-Nano or DOTAP-film was mixed with OVA antigen and was immunized under Glu/PBS buffer conditions into mice. DOTAP-Nano induced stronger T-cell responses than DOTAP-film in both CD8^+^ T-cell (class I) and CD4^+^ T-cell (class II) responses (Fig 2A). DLS measurement revealed that DOTAP-Nano itself is approximately 40 nm, and DOTAP-film is approximately 100 nm. In terms of OVA interaction, both DOTAP-Nano and DOTAP-film showed similar interactions as expected (Fig 2B). These results indicated that NanoAssemblr-formulated DOTAP-Nano has better adjuvanticity than DOTAP-film even though these two DOTAP preparations showed similar interactions with OVA antigen. This result shows that particle size is an important factor for the adjuvanticity of DOTAP particles.

### DOTAP-Nano plus Type-A CpG D35 induced better overall T-cell responses than other Type-B, -C, and -P CpG ODNs

**Fig 3:**
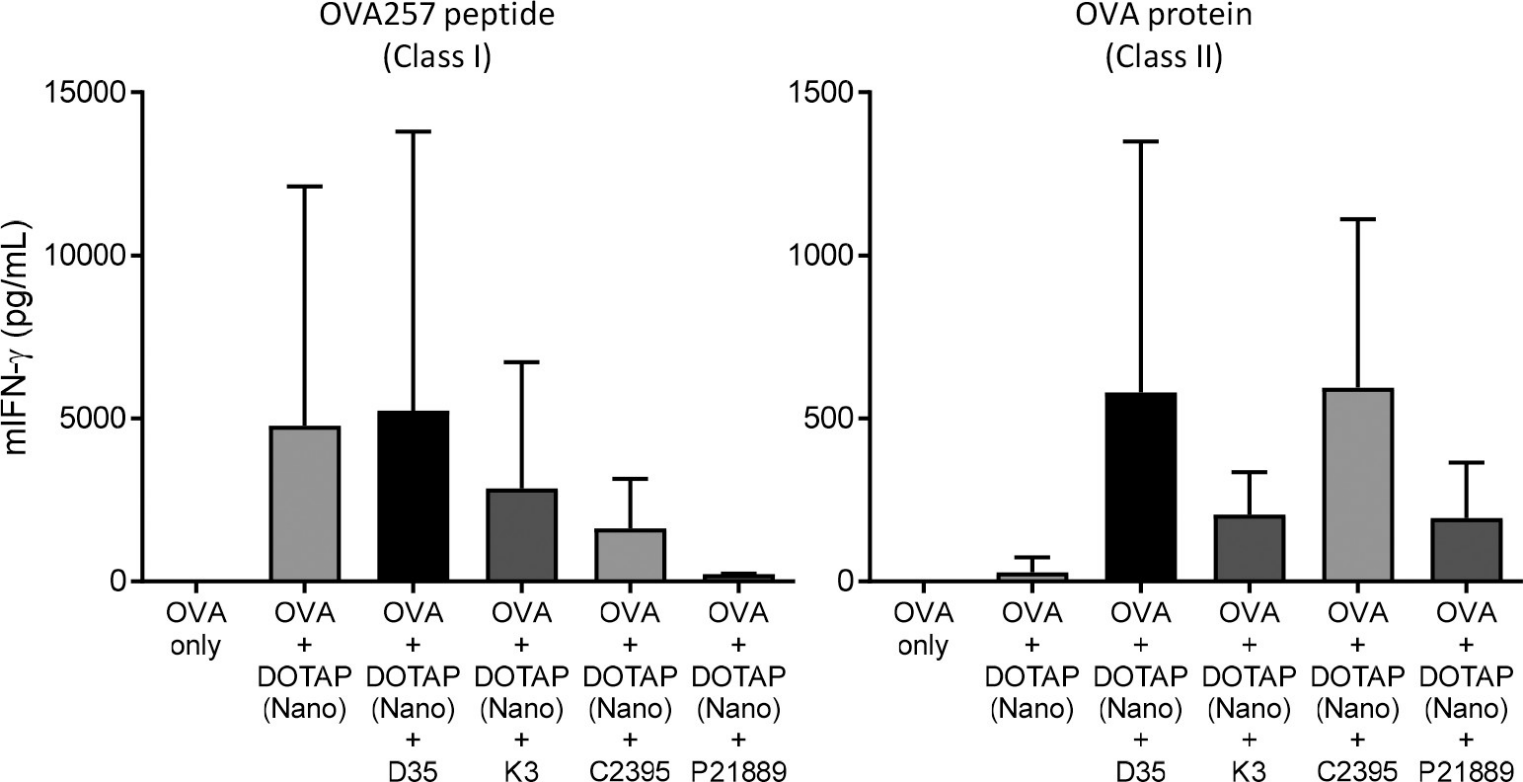
Addition of Type-A CpG further enhanced the adjuvanticity of DOTAP-Nano. C57BL/6J mice were immunized with OVA (10 μg), OVA plus DOTAP (100 μg), and OVA/DOTAP plus the indicated CpG adjuvant (10 μg) at the tail base. After seven days, splenocytes were collected and stimulated with OVA257-264 peptide and OVA protein. Twenty-four hours later, mouse IFN-gamma in the culture supernatant was measured by ELISA. The bar graph indicates the mean ± SD of three mice per group.

DOTAP has also been used as a carrier of CpG ODNs and is known to enhance CpG-mediated adjuvanticity. Four different types of CpG ODN, namely, Type-A, Type-B, Type-C, and Type-P, have been reported [35, 36], and the outcome of their combinations with DOTAP sometimes differed depending on the CpG type [27]. Therefore, each type of CpG ODN was added into the DOTAP-Nano/OVA complex, and the T-cell responses induced by adjuvanticity were examined. We chose D35, K3, C2395, and P21889 as representatives of each type of CpG ODN. Mice were immunized with a mixture of DOTAP-Nano/OVA with each CpG ODN, and the OVA-specific T-cell responses were determined seven days later. OVA-specific CD8^+^ T-cell responses were strongly induced by DOTAP-Nano/OVA and DOTAP-Nano/OVA plus D35. DOTAP-Nano/OVA plus other types of CpG including K3, C2395, and P21889 were relatively ineffective (Fig 3). In the case of P21889, the addition of this CpG ODN was even suppressive, particularly for the induction of CD8^+^ T-cell responses (Fig 3; right). OVA-specific CD4^+^ T-cell responses were increased by the addition of any type of CpG ODNs (Fig 3; left). However, among them, the addition of D35 or C2395 induced relatively stronger CD4^+^ T-cell responses (Fig 3; left). These results suggested that the addition of Type-A CpG (such as D35) to DOTAP-Nano/OVA induced comparable CD8^+^ and better CD4^+^ T-cell responses than DOTAP-Nano/OVA only.

### Antigen interaction with DOTAP-Nano is essential even in combination with CpG ODNs

The adjuvanticity of DOTAP-Nano is dependent on the interaction with antigen (Fig 1), and the adjuvanticity of DOTAP-Nano is further enhanced by adding D35, which is a Type-A CpG ODN (Fig 3). Considering that antigen–adjuvant interaction is not always necessary for some immune-potentiating particle adjuvants including advax [37], AS03 [38], and MF59 [39], we also examined whether the addition of D35 into the OVA antigen-noninteracting DOTAP-Lipo in MES-buffered saline showed adjuvanticity.

**Fig 4:**
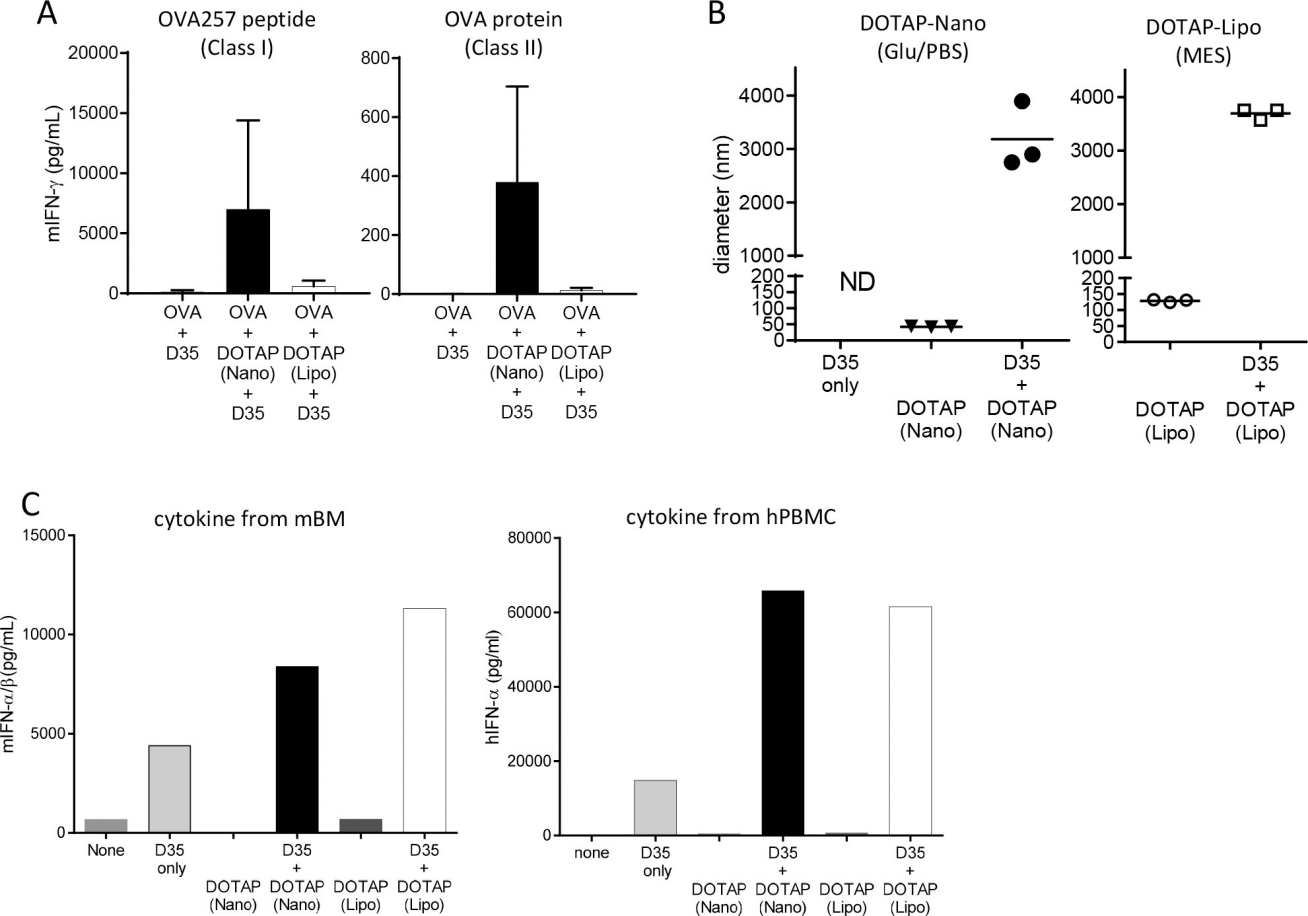
Antigen interaction is essential for the adjuvanticity of DOTAP-Nano plus D35. **(A)** C57BL/6J mice were immunized with OVA (10 μg) and DOTAP (100 μg) with D35 at the tail base. After seven days, splenocytes were collected and stimulated with OVA257-264 peptide and OVA protein. Twenty-four hours later, mouse IFN-gamma in the culture supernatant was measured by ELISA. The bar graph indicates the mean ± SD of three mice per group. **(B)** Particle size was measured by DLS. The sizes of D35 only, lipid particles only, and lipids mixed with D35 were measured under the indicated buffer conditions. An increase in particle size indicates that the particle interacts with D35. Each dot indicates one measurement. **(C)** Mouse BM (left panel) or human PBMCs (right panel) were incubated with D35 (6.3 μg/mL) mixed with DOTAP-Nano or DOTAP-Lipo. DOTAP-Nano and DOTAP-Lipo only were also included as controls. Twenty-four hours later, the mouse IFNα/β concentration in the supernatant was measured by B16-Blue reporter cell assay. Human IFN-α concentration was measured by ELISA.

OVA immunization with DOTAP-Lipo plus D35 (in MES-buffered saline; under these conditions, DOTAP does not interact with OVA, as shown in Fig 1) did not induce T-cell responses compared with DOTAP-Nano plus D35 (in Glu/PBS buffer; interacting with OVA) (Fig 4A). The DLS evaluation of the interaction between lipid and D35 showed that D35 similarly interacted with DOTAP-Nano and DOTAP-Lipo (Fig 4B). To confirm that these lipids and nucleic acid interactions are functional, mouse BM cells or human PBMCs were stimulated with D35 only or in combination with D35 plus either DOTAP-Nano or DOTAP-Lipo. Both DOTAP-Nano and DOTAP-Lipo worked as efficient systems for delivering D35 into mouse and human cells, thus resulting in increased IFN-α production (Fig 4C). These results suggested that both DOTAP-Nano and DOTAP-Lipo interacted with D35 and enhanced D35-mediated IFN-α production (which is a potent immunostimulatory cytokine). However, the D35-mediated induction of IFN-α still did not result in the strong induction of a T-cell response in the case of DOTAP-Lipo adjuvant (in MES-buffered saline; not interacting with OVA); this suggested that the interaction between DOTAP and antigen is essential even when D35 is included in DOTAP particle adjuvant.

### DOTAP-Nano did not work as an adjuvant for noninteracting HEL antigen

**Fig 5:**
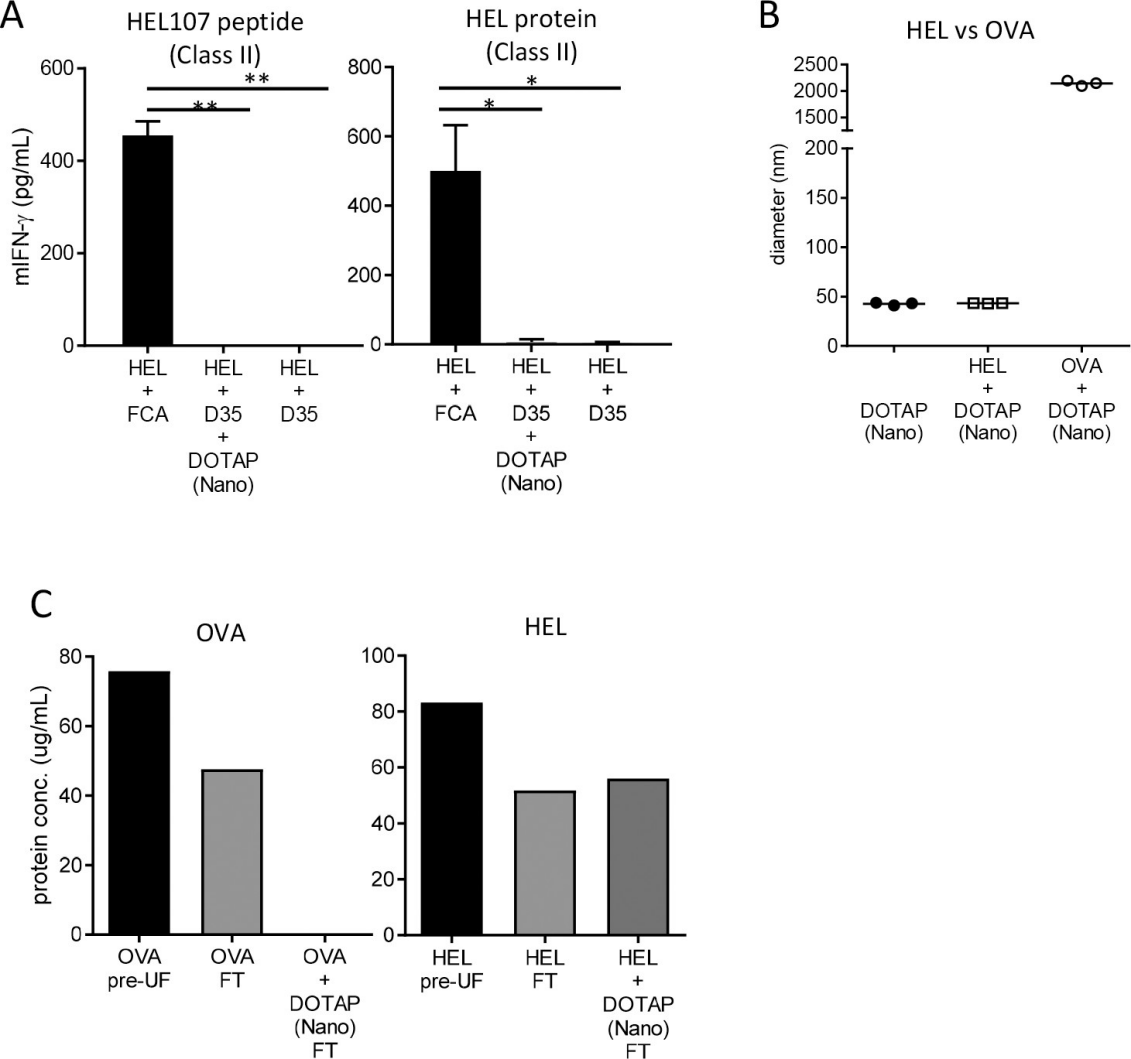
DOTAP-Nano plus D35 did not show adjuvanticity for HEL antigen. **(A)** BALB/cByJ mice were immunized with HEL (10 μg) and DOTAP (100 μg) plus D35 (10 μg) at the tail base. After seven days, splenocytes were collected and stimulated with HEL 107–116 peptide or HEL protein for the CD4^+^ T-cell responses. Twenty-four hours later, mouse IFN-γ in the culture supernatant was measured by ELISA. The bar graph indicates the mean ± SD of three mice per group. **(B)** The particle size was measured by DLS. DOTAP-Nano was mixed with HEL or OVA. DOTAP-Nano and HEL did not show any interactions. Each dot indicates one measurement. **(C)** OVA or HEL was mixed with DOTAP-Nano under Glu/PBS buffer conditions. The mixtures were ultrafiltrated to separate lipid binding antigen and free antigen in the flow through (FT). The protein concentration in the FT solution was measured. In the case of OVA mixed with DOTAP-Nano, no free OVA was detected in the FT solution (left graph). In the case of HEL, almost all HEL was detected in the FT (right graph).

To further investigate the necessity of antigen–lipid interaction, we tested HEL as another model antigen. The isoelectric point of HEL is approximately 11 (i.e., HEL antigen is positively charged in physiological pH solution), thus suggesting that HEL and DOTAP do not show electrostatic interaction. BALB/c mice were similarly immunized with HEL with DOTAP-Nano plus D35 in Glu/PBS, and CD4^+^ T-cell responses against HEL107-116/I-E^d^ were evaluated by IFN-γ ELISA. HEL plus Freund’s complete adjuvant immunization induced IFN-γ responses, whereas HEL with DOTAP-Nano plus D35 did not induce CD4^+^ T-cell responses (Fig 5A). The DLS measurement of the interaction between lipid and antigen also confirmed that HEL antigen did not interact with DOTAP-Nano, whereas OVA antigen interacted with DOTAP-Nano in Glu/PBS (Fig 5B). To further clarify whether these interactions are physically stable, OVA or HEL was mixed with DOTAP-Nano, and then free antigen and lipid/antigen complex were separated by UF. In the case of OVA, FT solution did not contain a detectable level of OVA protein (Fig 5C; left). In the case of HEL, almost 100% of HEL protein was detected in the FT (Fig 5C; right). These results suggested that DOTAP-Nano plus D35 adjuvanticity was completely dependent on the presence of a physically stable electrostatic interaction with antigen protein.

### DOTAP-Nano plus D35 induced detectable T-cell responses upon immunization with a relatively low dose of antigen

**Fig 6:**
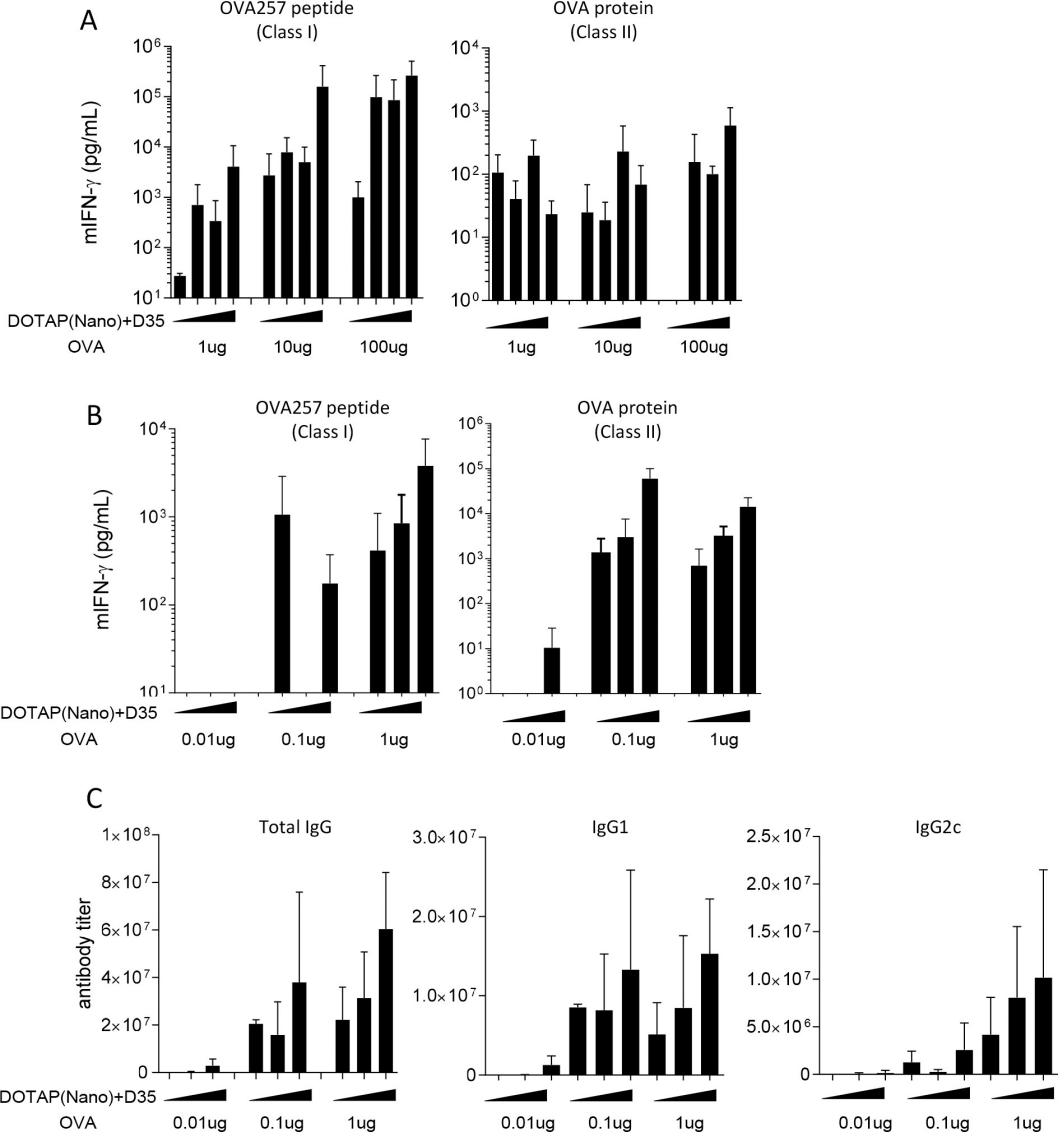
DOTAP-Nano plus D35 induces T-cell responses upon immunization with a small amount of OVA antigen. **(A)** C57BL/6J mice were immunized with the indicated combination of OVA and adjuvant (DOTAP-Nano plus D35) at the tail base. After seven days, splenocytes were collected and stimulated with OVA257-264 peptide and OVA protein for CD8^+^ and CD4^+^ T-cell responses, respectively. Twenty-four hours later, mouse IFN-gamma in the culture supernatant was measured by ELISA. The bar graph indicates the mean ± SD of three mice per group. **(B)** C57BL/6J mice were immunized three times at a two-week interval, with the indicated combination of OVA and adjuvant (DOTAP-Nano plus D35) at the tail base. Seven days after the last immunization, splenocytes were collected and stimulated with OVA257-264 peptide and OVA protein for CD8^+^ and CD4^+^ T-cell responses, respectively. Twenty-four hours later, mouse IFN-gamma in the culture supernatant was measured by ELISA. The bar graph indicates the mean ± SD of three mice per group. **(C)** Serum was collected seven days after the last immunization from C57BL/6J mice, as shown in Fig 6B. OVA-specific antibody responses were evaluated as described in the method section. The bar graph indicates the mean ± SD of three mice per group.

Finally, we tested more practical aspects of using the DOTAP-Nano-based adjuvant. We chose DOTAP-Nano plus D35 adjuvant in this experiment and examined the combination of different OVA antigen doses (1, 10, and 100 μg) and different D35 doses (1, 10, 20, and 30 μg; notably, the ratio of D35:DOTAP weights was kept at 1:10, and DOTAP was proportionally increased as the amount of D35 increased) for the induction of T-cell responses. Mice were immunized, and splenocytes were evaluated seven days later for the induction of OVA-specific T-cell responses. OVA257-264 peptide–specific MHC class I–restricted CD8^+^ T-cell responses were induced in a manner that is dependent on both antigen and adjuvant (DOTAP-Nano plus D35). Increased amounts of antigen and adjuvant resulted in better CD8^+^ T-cell responses (Fig 6A; left). In CD4^+^ T-cell responses, dose proportionality was not clearly observed among the examined combinations (Fig 6A; right). These results suggested that DOTAP-Nano plus D35 efficiently induced T-cell responses in mice even with a relatively small amount of antigen such as 1 μg of OVA and only a single immunization.

DOTAP-Nano plus D35 adjuvant was also examined in the prime-boost protocol. Mice were immunized with the combinations of different OVA doses (0.01, 0.1, and 1 μg) and different DOTAP-Nano plus D35 adjuvant doses (1, 5, and 10 μg as the amounts of D35) three times at two-week intervals. In this immunization protocol, DOTAP-Nano plus D35 adjuvant induced both CD8^+^ and CD4^+^ T-cell responses even upon immunization with 0.1 μg of OVA antigen (Fig 6B). We also examined OVA-specific antibody responses in this prime-boost immunization protocol. OVA-specific total IgG, IgG1, and IgG2c were clearly detected in a manner that is dependent on both antigen and adjuvant doses (Fig 6C). Among these antibody responses, IgG2c responses required relatively strong antigen and adjuvant dose immunization. Taken together, these results suggested that DOTAP-Nano plus D35 is a highly efficient adjuvant for inducing both CD8^+^ and CD4^+^ T-cell responses with a relatively small amount of antigen and that it is capable of inducing efficient antibody responses with the prime-boost protocol.

## Discussion

DOTAP is one of the most popularly used cationic lipid for vaccine formulation and development studies ([18–21, 24–27]. However, what physicochemical factors are important for the DOTAP mediated biological effects is not fully understood yet. In this study, we examined the adjuvanticity of DOTAP nanoparticles, particularly for the induction of T-cell responses. Our results suggested that there are three important factors affecting the adjuvanticity of DOTAP nanoparticles: 1) preparation method, 2) size, and 3) interaction with antigen.

Although the underlying detailed mechanisms need to be examined extensively in future works, the relatively small DOTAP nanoparticle preparations obtained using a microfluidic processor such as NanoAssemblr showed better adjuvant activity than those obtained using conventional particle preparation, such as the hydration film method. Although DOTAP-Nano and DOTAP-film showed similar lipid–OVA interactions, the adjuvanticity was still better in DOTAP-Nano (Fig 2). Microfluidics has been commonly used to prepare siRNA-containing lipid nanoparticle formulations but has not really been examined as a method for preparing lipid nanoparticle adjuvants. This study demonstrated that microfluidic preparation itself can affect the adjuvanticity of DOTAP nanoparticles. Furthermore, other factors such as interaction with antigen (Fig 2A and 2B) and differences in buffer conditions (Fig 1E; right) were also important.

The relatively small particle size (<100 nm) prepared by microfluidics could influence the biodistribution of DOTAP nanoparticles. Currently, experiments examining the biodistribution in tissue, such as in draining lymph nodes, are ongoing. However, thus far, we have not observed any apparent differences of biodistribution between the small and large preparations of DOTAP (unpublished data).

Another important factor is the interaction between DOTAP nanoparticles and antigens. The difference in DOTAP–antigen interaction depending on the buffer conditions resulted in clear differences in the induction of a T-cell response by protein vaccine antigen. DOTAP nanoparticles did not induce strong T-cell responses without DOTAP–antigen interaction (Fig 1). We also confirmed this result by using different antigens of HEL, which did not interact with DOTAP (Fig 5B and 5C), and the HEL DOTAP adjuvant combination did not result in the induction of T-cell responses (Fig 5A). Interestingly, T-cell responses against HEL antigen were not observed in this case even when using DOTAP-Nano plus D35. DOTAP-Nano plus D35 enhanced IFN-α induction in mouse and human cells and has been shown to stimulate the induction of T-cell responses, including cross-presentation [40–42]. In this case, strong immunostimulatory cytokine induction or inflammatory responses could compensate for the lack of lipid–antigen interaction and result in the induction of a T-cell response, which has been shown in other originally antigen-noninteracting adjuvants such as advax [37], AS03 [38], and MF59 [39]. These adjuvants do not require antigen–adjuvant interactions to induce T-cell responses. However, in the case of DOTAP-Nano plus D35, the T-cell response agonist HEL was also dependent on DOTAP–antigen interaction, thus suggesting that antigen interaction is almost obligatory in the case of DOTAP nanoparticle adjuvant.

In this study, we did not sufficiently investigate the following points, which are considered important in understanding the mechanisms of induction of DOTAP-Nano (plus D35)-mediated T-cell responses: 1) innate immune mechanisms of DOTAP-Nano recognition because DOTAP-Nano itself induced strong CD8^+^ T-cell responses without D35 (Fig 3) and 2) the size-dependent differences in biodistribution of DOTAP at the cellular and tissue levels. These two points are generally important in understanding the mechanism of action of particle adjuvants. Therefore, future experiments should be performed to understand the mechanism of action of DOTAP-Nano adjuvant.

In summary, we demonstrated that antigen–adjuvant interaction is essential for the induction of T-cell responses by DOTAP-Nano adjuvant even if a TLR ligand such as DOTAP-Nano plus D35 is included. Furthermore, interestingly, the microfluidic preparation method itself and the resulting small DOTAP nanoparticles (<100 nm) showed better T-cell response–inducing adjuvanticity, thus suggesting that microfluidic-prepared lipid particles are a promising adjuvant candidate for future T-cell response–inducing vaccines.

## Supporting information

S1 Fig Particle characterizations. Particle size was measured by DLS. The sizes of DOTAP-particle only, and mixed with antigen protein or D35 were measured under indicated buffer conditions. The value of Polydispersity index (PdI) indicates the mean ± SD of three times measurements.
(TIF)

S2 Fig Physical complex formation between DOTAP-nano and different types of CpGs. A: Particle size was measured by DLS. The sizes of DOTAP-Nano only, and mixed with indicated CpG were measured under Glu/PBS buffer conditions. An increase in particle size indicates that the particle interacts with CpG. Each dot indicates one measurement.
B: D35 and other indicated CpG was mixed with DOTAP-Nano under Glu/PBS buffer conditions. The mixtures were ultrafiltrated to separate lipid binding DNA and free DNA in the flow through (FT). The DNA concentration in the FT solution was measured. No free DNA was detected in the FT solutions from all samples, indicating that almost 100% physical complex formation between any types of CpG when mixed with DOTAP-Nano.
(TIF)
