# Supplementary figures and images for: Microfluidic-prepared DOTAP nanoparticles induce strong T-cell responses in mice
Source: PLoS One. 2020 Jan 24;15(1):e0227891. doi: 10.1371/journal.pone.0227891 (PMC6980563; doi:10.1371/journal.pone.0227891)

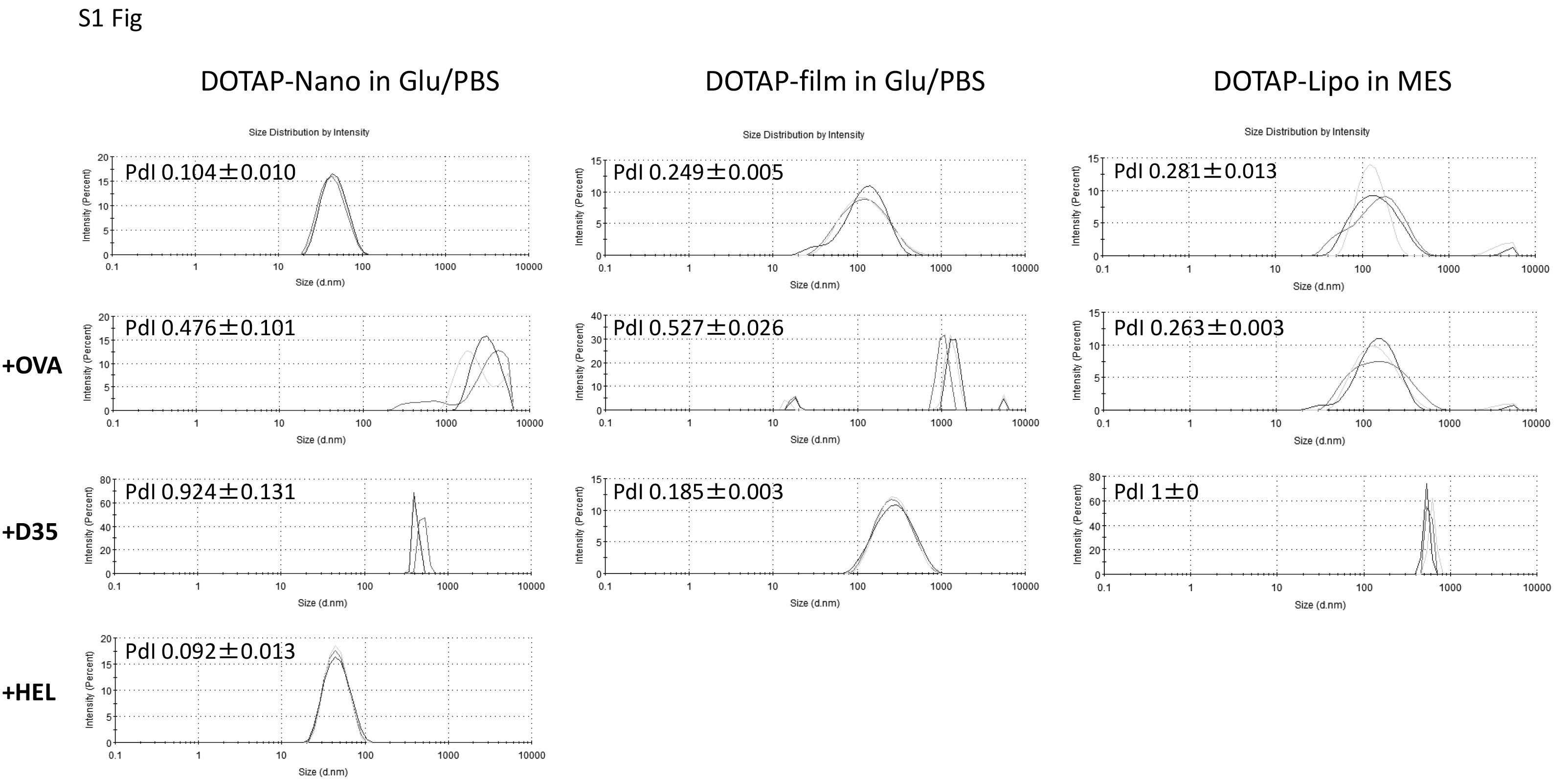

Supplement: S1 Fig — Particle size was measured by DLS. The sizes of DOTAP-particle only, and mixed with antigen protein or D35 were measured under indicated buffer conditions. The value of Polydispersity index (PdI) indicates the mean ± SD of three times measurements. (TIF) [file pone.0227891.s001.tif]

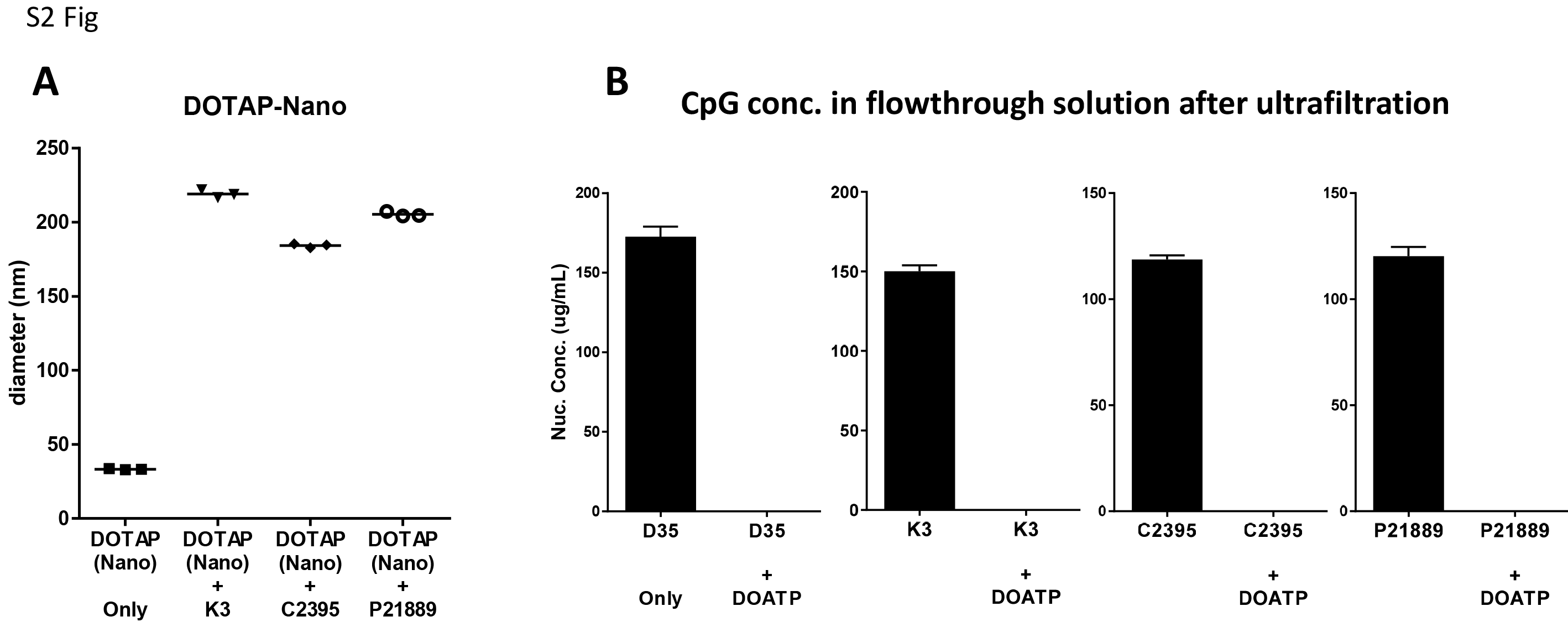

Supplement: S2 Fig — A: Particle size was measured by DLS. The sizes of DOTAP-Nano only, and mixed with indicated CpG were measured under Glu/PBS buffer conditions. An increase in particle size indicates that the particle interacts with CpG. Each dot indicates one measurement. B: D35 and other indicated CpG was mixed with DOTAP-Nano under Glu/PBS buffer conditions. The mixtures were ultrafiltrated to separate lipid binding DNA and free DNA in the flow through (FT). The DNA concentration in the FT solution was measured. No free DNA was detected in the FT solutions from all samples, indicating that almost 100% physical complex formation between any types of CpG when mixed with DOTAP-Nano. (TIF) [file pone.0227891.s002.tif]
